# The sigmoid volvulus: surgical timing and mortality for different clinical types

**DOI:** 10.1186/1749-7922-5-1

**Published:** 2010-01-13

**Authors:** Roberto Cirocchi, Eriberto Farinella, Francesco La Mura, Umberto Morelli, Stefano Trastulli, Diego Milani, Micol S Di Patrizi, Barbara Rossetti, Alessandro Spizzirri, Ioanna Galanou, Konstandinos Kopanakis, Valerio Mecarelli, Francesco Sciannameo

**Affiliations:** 1 Department of General Surgery, St Maria Hospital, Terni, University of Perugia, Italy; 2 III Department of Surgery, Attikon University Hospital, Athens, Greece

## Abstract

**Background:**

In western countries intestinal obstruction caused by sigmoid volvulus is rare and its mortality remains significant in patients with late diagnosis. The aim of this work is to assess what is the correct surgical timing and how the prognosis changes for the different clinical types.

**Methods:**

We realized a retrospective clinical study including all the patients treated for sigmoid volvulus in the Department of General Surgery, St Maria Hospital, Terni, from January 1996 till January 2009. We selected 23 patients and divided them in 2 groups on the basis of the clinical onset: patients with clear clinical signs of obstruction and patients with subocclusive symptoms. We focused on 30-day postoperative mortality in relation to the surgical timing and procedure performed for each group.

**Results:**

In the obstruction group mortality rate was 44% and it concerned only the patients who had clinical signs and symptoms of peritonitis and that were treated with a sigmoid resection (57%). Conversely none of the patients treated with intestinal derotation and colopexy died. In the subocclusive group mortality was 35% and it increased up to 50% in those patients with a late diagnosis who underwent a sigmoid resection.

**Conclusions:**

The mortality of patients affected by sigmoid volvulus is related to the disease stage, prompt surgical timing, functional status of the patient and his collaboration with the clinicians in the pre-operative decision making process. Mortality is higher in both obstructed patients with generalized peritonitis and patients affected by subocclusion with late diagnosis and surgical treatment; in both scenarios a Hartmann's procedure is the proper operation to be considered.

## Background

A volvulus is an abnormal twisting of the bowel on its mesenteric axis greater than 180 degrees [1], which produces an obstruction of the intestinal lumen and mesenteric vessels. Only a satisfactorily long mesenteric axis, as in the case of sigmoid colon, allows this torsion. The predisposing factors for the sigmoid volvulus are indeed the length of the sigmoid colon and the colon distension due to chronic constipation. The trigger factor causing the twisting of the sigmoid colon, maximally distended by the faecal impaction in constipated patients, is a quick emptying of the terminal faecal column portion in the sigma-rectum [2].

The sigmoid volvulus incidence is constantly reducing. At the beginning of the XX century, in the Guibè's record of occurrences [3], volvulus represented 16,9% of intestinal occlusions. Nowadays its incidence has considerably decreased and sigmoid volvulus is a rare event. Particularly in North America and Europe it represents 3,7-6% of all intestinal occlusions and it usually occurs in elderly patients with a greater incidence in the 8^th ^decade [4]. Conversely in other countries this pathology still shows a higher incidence: 24% in East India [5], 40% in North India [6], 32% in Iran [7], 31% in Zimbabwe [8], 54% in Ethiopia [9], 33% in Sudan [10] and 99% in Nigeria [11].

Although in western countries intestinal obstruction caused by sigmoid volvulus is rare, its mortality remains significant in patients with a late diagnosis [12]. The aim of this work is to assess which are the results of different surgical timings and procedures performed in the different clinical presentations of this disease.

## Methods

We realized a retrospective case note review of patients treated surgically for a sigmoid volvulus in the Department of General Surgery, St Maria Hospital, Terni, from January 1996 till January 2009.

We included in this study a group of 23 patients (15 men and 8 women), which were diagnosed at the Emergency Department with abdominal pain and obstructive symptoms and then admitted into other Departments for treatment. Nine patients were primarily admitted into the surgery unit with intestinal obstruction symptoms, while 14 patients were admitted for a subocclusion (8 patients were admitted in a medical unit and 6 patients in the surgery division). The patients were divided in 2 groups on the basis of the clinical onset: obstructed patients (9 patients) and subocclusive patients groups (14 patients) according to the following criteria: obstructed patients had abdominal distension with no flatus, tenderness and a clearly positive plain abdominal X-ray, whereas subocclusive patients had no flatus, moderate abdominal distension, and a doubtful plain abdomen X-ray.

All patients underwent clinical examination and an abdominal X-ray. We identified patients affected by the comorbidities included into Satariano's co-morbidity index [13], uncooperative patients with degenerative and cognitive diseases, patients with clinical signs of peritonitis and patients with a diagnostic abdominal X-ray for sigmoid volvulus or intestinal occlusion. We assessed 30-day postoperative mortality relating it to the surgical timing and treatment employed for each group.

## Results

**Figure 1:**
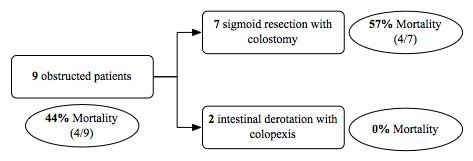
**Surgical timing and mortality in obstructed patients group**.

The mean age of patients with obstruction was 76 years (69-85 years). In this group 4 patients were affected by >2 comorbidities and 5 patients by <2 comorbidities. Three patients were uncooperative and 2 of these were bed-bound. Four patients had clinical signs and symptoms of peritonitis and ileus, showing a diagnostic abdominal X-ray for sigmoid volvulus or intestinal occlusion, while the 5 remaining patients presented clinical and radiological signs of occlusion, but no clinical signs of peritonitis (Table 1). All the patients underwent emergency surgery; we performed a sigmoid resection in the 4 patients with clinical signs and symptoms of peritonitis and in 3 out of the 5 patients showing only clinical and radiological signs of occlusion, while an intestinal derotation with colopexy was performed in the 2 remaining patients. Mortality in the occlusive patients group was 44% (4/9), but if we also consider the obstructed patients with clinical signs and symptoms of peritonitis that were treated with a sigmoid resection, mortality rises up to 57% (4/7). Conversely none of the patients undergoing the intestinal derotation and colopexy died (Figure 1).

**Table 1:** Clinical characteristics of the patients at admission time.

	Obstructed patients group	Subocclusive patients group	Total
**Patients**	9	14	23
**Male/Female**	7/2	8/6	15/8
**Mean age**	76 years	81 years	79 years
**Comorbidities ≤ 2**	5	2	7
**Comorbidities >2**	4	12	16
**Uncollaborative**	3	9	12
**Bed-bound at admission time**	2	4	6
**Peritonitis**	4	0	4
**Diagnostic abdominal X-ray**	9	0	9

Mean age of the subocclusive patients group was 81 years (69-86 years). Twelve patients had >2 comorbidities and 2 patients had <2 comorbidities. Nine were uncooperative patients and 4 of these were bed-bound. At admission time none of them showed clinical signs of peritonitis neither a diagnostic abdominal X-ray for sigmoid volvulus nor intestinal occlusion (Table 1). The clinical presentation was not specific, being characterized by abdominal distension, cramp-like abdominal pain without fever, nausea and no flatus. Subsequently 6 of these patients underwent a CT scan, while the other 8 patients included in this group, were treated with medical therapy (fluid and electrolyte restoration, flatus tube, NGT if vomit and analgesia) without performing any further investigation. The different therapeutic approach mostly depended on the different physicians involved in the early clinical evaluation.

**Figure 2:**
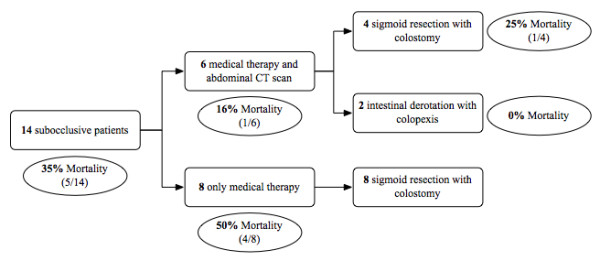
**Surgical timing and mortality in subocclusive patients group**.

An early diagnosis was only possible in the patients who underwent a CT scan, which showed typical signs of sigmoid occlusion. A sigmoid resection was performed in 4 patients and an intestinal derotation with colopexy was performed in 2 patients. One of the patients treated with sigmoid resection died on the 4th postoperative day. Mortality in the subocclusive patients with earlier CT diagnosis of volvulus was 16% (1/6). On the other hand in the 8 patients treated conservatively without CT scan, clinical and radiological signs of occlusion occurred within 48-72 hours, while 4 of them developed clinical signs and symptoms of peritonitis. For this reason all of them underwent a sigmoid resection in emergency. Four of them died within the 7th postoperative day (50%). Mortality in the subocclusive patients group with delayed diagnosis was 50% (4/8) (Figure 2).

In the subocclusive patients group mortality was 35% (5/14), but if we consider those patients who underwent a sigmoid resection, mortality increased up to 41% (5/12) and to 50% (4/8) in those patients with a delayed diagnosis.

In this series a colostomy was performed in all the patients treated with sigmoid resection (Hartmann's procedure) and none of them had restorative surgery afterwards.

The minor surgical complications, which occurred in both groups, were wound infection (3 patients) and transitory renal failure (2 patients).

## Discussion

Sigmoid volvulus can be divided in 2 clinical types with different onset and natural history [14]: the acute fulminating type (obstructed patients) and the subacute progressive one (subocclusive patients). The first kind is characterized by a sudden onset with abdominal pain, often localized in the umbilical region, early vomiting, abdominal tenderness, constipation and marked physical prostration. Gangrene usually develops early and perforation and shock may appear quickly. Whereas the subacute progressive form is characterized by an insidious onset and progression and it frequently occurs in older patients. It often shows an unspecific clinical presentation characterized by widespread cramp-like abdominal pain, sometimes localized in the left abdominal quadrants. Fever and vomiting are rare at the beginning.

An early diagnosis and management are crucial in both clinical types allowing the treatment of the sigmoid volvulus before the appearance of the twisted loop necrosis, and avoiding further complications. The ischemia is often due to an abnormal and prolonged distension of the twisted loop rather than to strangulation and for this reason ischemic necrosis can appear in a later stage [15]. When an on-call endoscopy team is available, it is advisable to perform a two-step management with a significant reduction of operative mortality. The first step is an endoscopic derotation followed by a sequent elective surgical correction by colopexy. The early diagnosis is more frequent in the patients with acute fulminating type because of specific clinical and radiological signs of occlusion and/or clinical signs of peritonitis, whereas it is often uncertain in those patients affected by the subacute progressive type because of its ambiguous and insidious onset and progression. Furthermore the subacute progressive type usually occurs in elderly patients who are often affected by several comorbidities and that are unable to collaborate. Nevertheless also in this patients group the possibility of achieving an early diagnosis remains fundamental as any delay may increase the mortality rate.

The prognosis of patients affected by sigmoid volvulus tightly depends on the disease stage, surgical timing and comorbidities. In fact the highest mortality rate is observed in the obstructed patients group, in those patients with clinical signs and symptoms of peritonitis and ileus who underwent Hartmann's procedure (57%). Mortality rate also results high in those patients belonging to the subocclusive patients group with late diagnosis and necessarily treated with Hartmann's (50%). Conversely, mortality reduces up to 16% in the patients affected by subocclusion with an early diagnosis achieved through CT scan. In these cases it is not always necessary to perform a sigmoid resection with colostomy, but, if sigmoid colon is still vital, a simple intestinal derotation with colopexy can be the right effective choice. Furthermore the prognosis is worse for the patients with advanced stages of disease and late diagnosis, as they are usually older, uncollaborative, bed-bound at admission and affected by several comorbidities (> 2). In opposition prognosis is more favourable in younger patients affected by minor comorbidities (< or = 2), being the diagnosis easier to achieve in these cases.

Although the data from our case series show that the Hartmann's procedure is associated with a higher postoperative mortality (57% in obstructed patients and 41% in the patients affected by subocclusion) than the intestinal derotation with colopexy (0% in both groups of patients), we do not retain that it represents an unsuccessful prognostic factor itself. Indeed this procedure is associated with an unfavourable prognosis as it is mostly performed in severe cases which are often associated with intestinal sigmoid necrosis.

The abdominal X-ray may show unspecific signs of sigmoid volvulus, but it is not able to offer an etiologic diagnosis. Indeed in 30-40% of the cases the abdominal X-ray is not diagnostic for sigmoid volvulus [16] because the transverse colon or small bowel distension can superimpose upon the sigmoid loops. Furthermore a redundant transverse colon or an obstructed small bowel loop may mimic a sigmoid volvulus [17,18]. Conversely CT scan allows to achieve a diagnosis even in the indeterminate cases [19-21] being particularly useful in the patients affected by intestinal subocclusion with ambiguous and insidious clinical onset and progression, and allowing an earlier diagnosis with a lower mortality.

The main limitation of this series is due to the fact that we analyzed patients with sigmoid volvulus treated with emergency surgery, while we excluded the majority of them being managed successfully with medical therapy; we also included patients in an advanced disease stage (ischemia/peritonitis). Therefore the advanced disease stage, the treatment performed in emergency and the elderly age of our population with a poor functional status could justify the high mortality rate that was detected.

## Conclusions

The mortality of patients with sigmoid volvulus treated surgically is closely related to the disease stage, a prompt surgical timing, the patient functional status and his collaboration with clinicians in order to define a correct diagnosis and treatment. For this reason mortality is higher in both obstructed patients with generalized peritonitis and patients affected by subocclusion with late diagnosis and undergoing surgery in advanced stages; in both cases an emergency Hartmann's procedure (57% and 50% mortality rate respectively) is to be considered.

However in both patients groups an early management is crucial in order to avoid necrosis of the twisted loop and the consequent mortality increase. In the subocclusive patients group an early diagnosis is even more fundamental because it allows a mortality reduction up to 16% in the patients investigated with CT scan. In this patients group the diagnosis of volvulus is more difficult because of its ambiguous and insidious clinical onset and progression. Furthermore subocclusive patients are usually older, uncollaborative, already bed-bound at admission and affected by several comorbidities. In this subset of patients the achievement of an early diagnosis through CT scan performance is strictly advised.

## Abbreviations

CT: computer tomography; NGT: naso-gastric tube.

## Competing interests

The Authors state that none of the authors involved in the manuscript preparation has any conflicts of interest towards the manuscript itself, neither financial nor moral conflicts. Besides none of the authors received support in the form of grants, equipment, and/or pharmaceutical items.

## Authors' contributions

All authors contributed equally to this work, read and approved the final manuscript.
